# Estimating the strength of bi-axially loaded track and channel cold formed composite column using different AI-based symbolic regression techniques

**DOI:** 10.1038/s41598-024-69241-6

**Published:** 2024-08-21

**Authors:** Ahmed M. Ebid, Mohamed A. El-Aghoury, Kennedy C. Onyelowe, Dina M. Ors

**Affiliations:** 1 Faculty of Engineering and Technology, Future University in Egypt, New Cairo, Egypt; 2 Department of Civil Engineering, Faculty of Engineering, Ain Shams University, Cairo, Egypt; 3 Department of Civil Engineering, Michael Okpara University of Agriculture, Umudike, Nigeria

**Keywords:** Cold-formed composite columns, Biaxial loading, Track and channel, Axial capacity, Symbolic regression, Civil engineering, Structural materials

## Abstract

Steel construction is increasingly using thin-walled profiles to achieve lighter, more cost-effective structures. However, analyzing the behavior of these elements becomes very complex due to the combined effects of local buckling in the thin walls and overall global buckling of the entire column. These factors make traditional analytical methods difficult to apply. Hence, in this research work, the strength of bi-axially loaded track and channel cold formed composite column has been estimated by applying three AI-based symbolic regression techniques namely (GP), (EPR) and (GMDH-NN). These techniques were selected because their output models are closed form equations that could be manually used. The methodology began with collecting a 90 records database from previous researches and conducting statistical, correlation and sensitivity analysis, and then the database was used to train and validate the three models. All the models used local and global slenderness ratios (λ, λc, λt) and relative eccentricities (ex/D, ey/B) as inputs and (F/Fy) as output. The performances of the developed models were compared with the predicted capacities from two design codes (AISI and EC3). The results showed that both design codes have prediction error of 33% while the three developed models showed better performance with error percent of 6%, and the (EPR) model is the simplest one. Also, both correlation and sensitivity analysis showed that the global slenderness ratio (λ) has the main influence on the strength, then the relative eccentricities (ex/D, ey/B) and finally the local slenderness ratios (λc, λt).

## Introduction

Cold-formed steel (CFS) sections play a crucial role in the construction industry due to their high strength-to-weight ratio, versatility, and ease of fabrication. These sections are manufactured by rolling or pressing thin steel sheets at room temperature, which imparts desirable mechanical properties without the need for additional heat treatment. The inherent strength and flexibility of CFS sections enable innovative architectural designs while ensuring structural integrity and safety. Additionally, their lightweight nature reduces transportation and handling costs, and their recyclability aligns with sustainable construction practices. CFS sections are particularly advantageous in applications such as wall framing, roof trusses, and floor systems, where their precision and uniformity contribute to efficient and economical building processes.

The application of cold-formed steel (CFS) sections as columns in structural systems offers several advantages and necessitates careful consideration of specific design parameters to ensure performance and safety. CFS columns are favored for their high strength-to-weight ratio, ease of fabrication, and adaptability to various architectural requirements. Usually they are used as competitive alternative for FRP traffic signs columns, light poles and low voltage power lines masts^1^. However, their slender nature makes them susceptible to different buckling modes, including local, distortional, and overall column buckling, which must be rigorously analyzed. The interaction of these buckling modes under axial and lateral loads requires detailed finite element modeling and stability analyses to predict and mitigate potential failure mechanisms. Additionally, the influence of residual stresses from the cold-forming process, material imperfections, and end conditions are critical factors that impact the column's load-bearing capacity and stability. The design must comply with established standards, such as those outlined by the American Iron and Steel Institute (AISI), to ensure that CFS columns can effectively support structural loads while maintaining safety and durability.

Designing cold-formed steel (CFS) sections necessitates a comprehensive understanding of several critical provisions to ensure structural performance and safety. Key considerations include local buckling, where thin elements may buckle under compressive stress before yielding, and distortional buckling, where a combination of bending and torsional deformations occurs. Engineers must also account for the effects of residual stresses induced during the cold-forming process, which can influence the section's strength and stability. Material properties such as yield strength and modulus of elasticity, typically derived from standardized testing, are fundamental in predicting the behavior of CFS sections under various loads. Furthermore, connection design is pivotal, as the integrity of the joints significantly impacts the overall structural performance.

## Literature review

Georgieva et al.^2^ conducted an experimental evaluation of the capacities of built-up columns composed of four distinct individual profiles: sigma, zed, channel, and track. The capacities of these proposed cross-sections were compared with the predicted results from the Direct Strength Method (DSM)^3–5^ and the European standards. Their findings indicated that the proposed columns were reliable and suitable for use in construction. Additionally, I. Georgieva et al.^6,7^ performed both experimental and numerical investigations on double-Z built-up members subjected to bending and compression loads. The study concluded that this cross-section was effectively utilized for primary members with spans between 10 and 18 m.

Li et al.^8^ conducted a series of axial compression tests on built-up columns composed of double channels, assembled using self-drilling screws to form box and I-shaped sections. Their findings indicated that box section columns effectively restrained distortional buckling and enhanced the column capacity. Additionally, the location of the fasteners influenced the failure mode and contributed to an increase in the ultimate strength of the columns.

The AISI^9^ established limitations on connector spacing to prevent the failure of individual components. According to these guidelines, the individual sections should be connected at the ends of the built-up member by a dense group of end connectors over a distance of at least 1.5 times the maximum width of the cross-section member. El Aghoury et al.^10,11^ conducted both experimental and numerical studies on the axial strength of double back-to-back cold-formed sigma columns. They utilized CUFSM^12,13^ to predict the elastic buckling loads and observed that variations in the spacing between interconnectors did not significantly affect the ultimate loads. Liu and Zhou^14^ tested built-up T-sections composed of three C-sections with two different cross-sectional dimensions under uniaxial compression loads.

Liu et al. observed that distortional failure occurred in short columns, while flexural–torsional buckling occurred in medium and long columns. In their study, Meza and Becque^15^ tested four different cross-section geometries of built-up fixed-ended stub columns and found that the behavior of connectors had no significant effect on the column strength. Additionally, Liao et al.^16^ investigated a multi-limbs built-up steel stub column with three different cold-formed cross-section shapes under compression load. They concluded that the axial load capacity of the column increased with decreasing width-to-thickness ratio of the plates, and the spacing between screws had a minimal effect on the ultimate load.

Anbarasu and Venkatesan^17^ conducted a study involving numerical simulations on built-up I-section columns, which were constructed using four cold-formed U-profiles. This configuration included two U-profiles arranged back-to-back to form the web, with the remaining two U-profiles used for each flange. Their research focused on evaluating the axial compressive loading performance. Anbarasu et al. proposed a modification to enhance the accuracy of the predicted ultimate strength utilizing the current direct strength method. Additionally, Roy et al.^18–20^ presented experimental and numerical investigations on the axial capacity of double-channel built-up cold-formed sections. Their findings indicated that existing design standards overestimated the axial load capacity by 53%, 15%, and 17% for gapped, face-to-face channel sections, and box sections, respectively.

El Aghoury et al.^21^ proposed a novel cold-formed built-up column cross-section consisting of double tracks and double channels. They recommended that the end spacing and intermediate interconnector spacing be set equal to twice and half of the cross-section depth, respectively. Numerical findings indicated that varying the interconnector spacing within these limits did not significantly affect the ultimate loads. Meza et al.^22^ experimentally investigated the behavior and ultimate capacity of cold-formed steel built-up columns. Their test results demonstrated that the spacing between connectors had a negligible effect on the ultimate load of the columns tested.

Li et al.^23^ conducted an experimental investigation on open built-up sections connected using self-drilling screws and subjected to eccentric compressive loads. The cross-section comprised double channels of cold-formed steel (CFS) with longitudinal stiffeners, and specimen lengths varied up to 1500 mm. Their study revealed that the axial load-moment interactive formulas generally underestimate the strength of cold-formed steel built-up open section beam-columns. Further analysis of the literature highlights that previous studies have primarily focused on the behavior of CFS built-up columns under axial loading, predominantly featuring cross-sections composed of channel, sigma, or zed sections. In contrast, there is a lack of research on CFS built-up columns subjected to uni-axial or bi-axial loads.

Harrat et al.^24^ and Reda et al.^25^, both experimentally investigated the behavior of CFS sections filled with concrete under eccentric loading condition. They concluded that filling the CFS sections with concrete minimized both local and global buckling and increased the axial capacity especially under small eccentricity loading condition.

Finally, it worth to mention the scientific efforts of many other researchers in implementing the (AI) techniques in optimizing the design of steel elements such as El-Aghoury et al.^26,27^ and Ebid et al.^28–30^, and in estimating the capacities of steel elements such as Ebid et al.^31–33^, and in structural engineering field generally such as Habib et al.^34–38^ and Shehadeh et al.^39–46^.

## Objective and novelty

The surveying earlier related researches showed a shortage in implementing recent (AI) technique in estimating the capacities of composite cold form columns especially those consists of the innovative sections such as track section. Hence, this research aims to fill this gap study by developing three (AI) based predictive models (GP, EPR and GMDH-NN) to estimate the strength of bi-axially loaded track and channel cold formed composite column considering local and global slenderness values and relative eccentricities.

## Methodology

The methodology employed in this study began with a review of relevant prior research to identify parameters significantly impacting column capacity. Additionally, sufficient experimental and numerical test results were collected to train and validate the developed (AI) models. Following data collection, three analyses were performed on the compiled database. First, a statistical analysis was conducted to ensure both training and validation subsets possessed identical statistical characteristics, while also establishing range limitations for each parameter. Second, a correlation analysis was undertaken to determine the interrelationships between the considered parameters and verify their independence. Finally, a sensitivity analysis was employed to quantify the impact of each input parameter on the output and identify the most significant factors.

**Figure 1:**
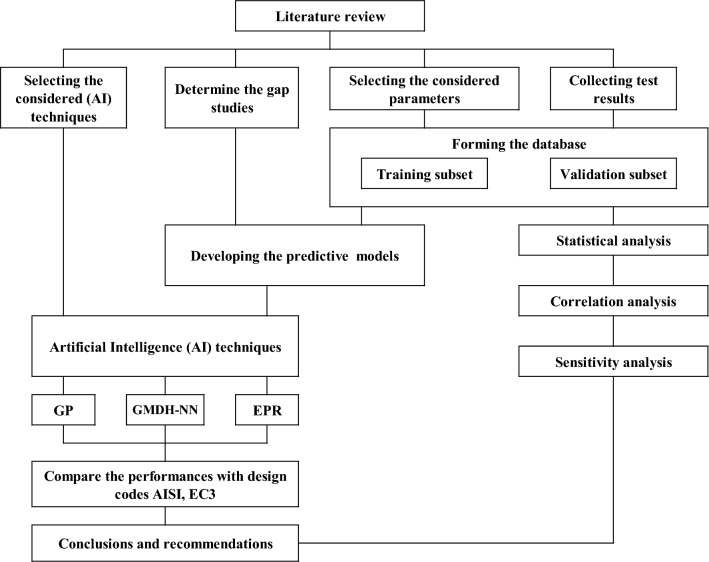
The considered methodology.

Subsequently, the three developed (AI) models were trained and validated using the collected data. The entirety of the study's findings were summarized in the conclusions section, along with any limitations encountered and recommendations for future work. Figure 1 provides a graphical representation of the employed methodology.

## The considered (AI) techniques

Three different symbolic regression techniques were used to predict the normalized strength (F/Fy) of bi-axial loaded track and channel cold formed composite column sing the collected database. These techniques are “Genetic programming” (GP), three models of “Group method of data handling Neural Network” (GMDH-NN) and “Evolutionary Polynomial Regression” (EPR). Flowcharts for the used techniques are presented in Fig. 4. All the three developed models were used to predict (F/Fy) using global and local slenderness ratios (λ, λc, λt) and relative eccentricities in both directions (ex/D, ey/B).

**Figure 2:**
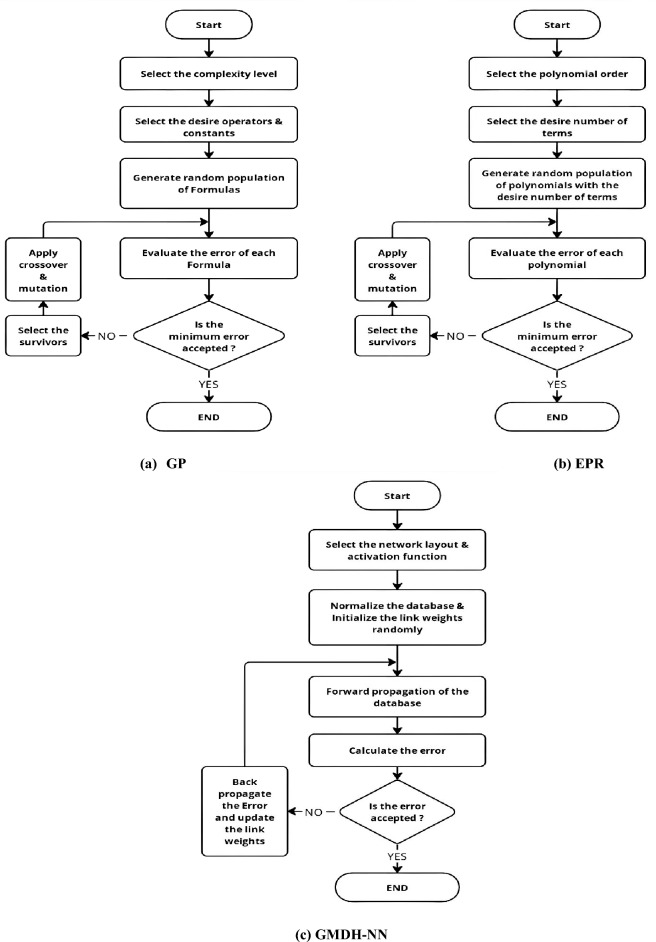
Flowcharts for different (AI)-based symbolic regression techniques.

Various metrics, including Sum of Squared Errors (SSE), Mean Absolute Error (MAE), Mean Squared Error (MSE), Root Mean Squared Error (RMSE), Error Percentage, Accuracy Percentage, and R-squared, were used to evaluate the prediction accuracy of all the developed models. The definitions for each metric are presented in Eqs. (1)–(6). Brief descriptions of these techniques are provided in the following paragraphs. Flowcharts for the three considered (AI) techniques are illustrated in Fig. 2.

1 $$MAE = \frac{1}{N}\sum\limits_{{i = 1}}^{N} {\left| y_{i} - {\hat{y}} \right|}$$

2 $$MSE = \frac{1}{N}\sum\limits_{{i = 1}}^{N} {\left( {y_{i} - \hat{y}} \right)^{2} }$$

3 $$RMSE= \sqrt{MSE}$$

4 $$Error \%=\frac{RMSE}{\widehat{y}}$$

5 $$Accurcy \%=1-Error \%$$

6 $${R}^{2}=1- \frac{\sum {\left({y}_{i}-\widehat{y}\right)}^{2}}{\sum {\left({y}_{i}-\overline{y }\right)}^{2}}$$

### Genetic programming (GP)

GP is a fascinating approach inspired by evolution to automatically create computer programs. Imagine a population of programs, each represented by a tree-like structure with functions and variables as its building blocks. These programs are then evaluated based on their ability to solve a specific problem. Just like in nature, the “fittest” programs, those performing well, are selected and used to create new generations. Through crossover, where parts of successful programs are combined, and mutation, where slight changes are introduced, new programs are born. Over multiple generations, GP iteratively refines the population, gradually evolving programs that can effectively tackle the given problem. This technique is particularly useful when the exact solution is unknown or complex, making it a valuable tool in various fields like engineering, finance, and even software design.

### Evolutionary polynomial regression (EPR)

EPR is a powerful technique for uncovering complex relationships between variables, particularly when a clear mathematical formula isn't readily available. It leverages the principles of evolutionary computing, mimicking the process of natural selection. Here's how it works: EPR starts with a pool of candidate equations, often simple polynomials. These equations are then evaluated based on their ability to fit the provided data. Through a series of iterations, similar to generations in evolution, "good" equations with high accuracy are retained and modified, while "bad" ones are discarded. Over time, these modifications lead to increasingly complex and accurate equations that effectively capture the underlying relationships within the data. This approach proves particularly useful in engineering and scientific fields where complex systems exhibit non-linear behaviors.

### Group method of data handling neural network (GMDH-NN)

GMDH-NN technique offers a powerful tool for modeling complex systems, particularly when the relationships between variables are unknown. Unlike traditional neural networks that require predefined structures, GMDH-NN builds its architecture automatically through a self-organizing process. It starts with simple mathematical functions applied to pairs of input variables. The outputs from these functions are then combined and fed into another layer of functions, creating progressively more complex models. An external criterion, such as prediction accuracy, is used to evaluate each model, and the GMDH-NN iteratively selects the best performing models to form the final network. This data-driven approach allows GMDH-NN to capture intricate relationships within the data and excel at tasks like prediction, pattern recognition, and system optimization.

## The collected database

**Figure 3:**
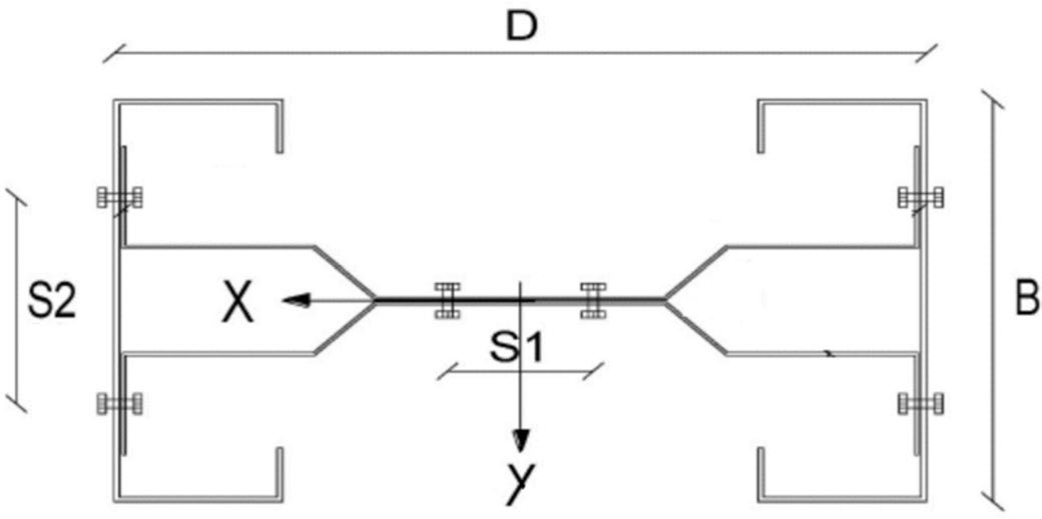
Typical cross section for track and channel cold formed composite column.

A total of ninety (90) records were collected for numerically tested samples of bi-axially loaded track and channel cold formed composite column^47^. Typical column cross section is shown in Fig. 3. Each record contains the following data:

- λ: Global slenderness ratio (Column height/minor radius of gyration)
- λc: Local slenderness ratio of channel (bolts spacing S2/channel thickness)
- λt: Local slenderness ratio of track (bolts spacing S1/track thickness)
- ex/D: Relative eccentricity in the major direction
- ey/B: Relative eccentricity in the minor direction
- F/Fy: Normalized Average normal stress at failure (Ult. load /Area)/yield stress

The collected records were divided into training set (70 records) and validation set (20 records). The Appendix includes the complete dataset.

### Statistical and correlation analysis

**Figure 4:**
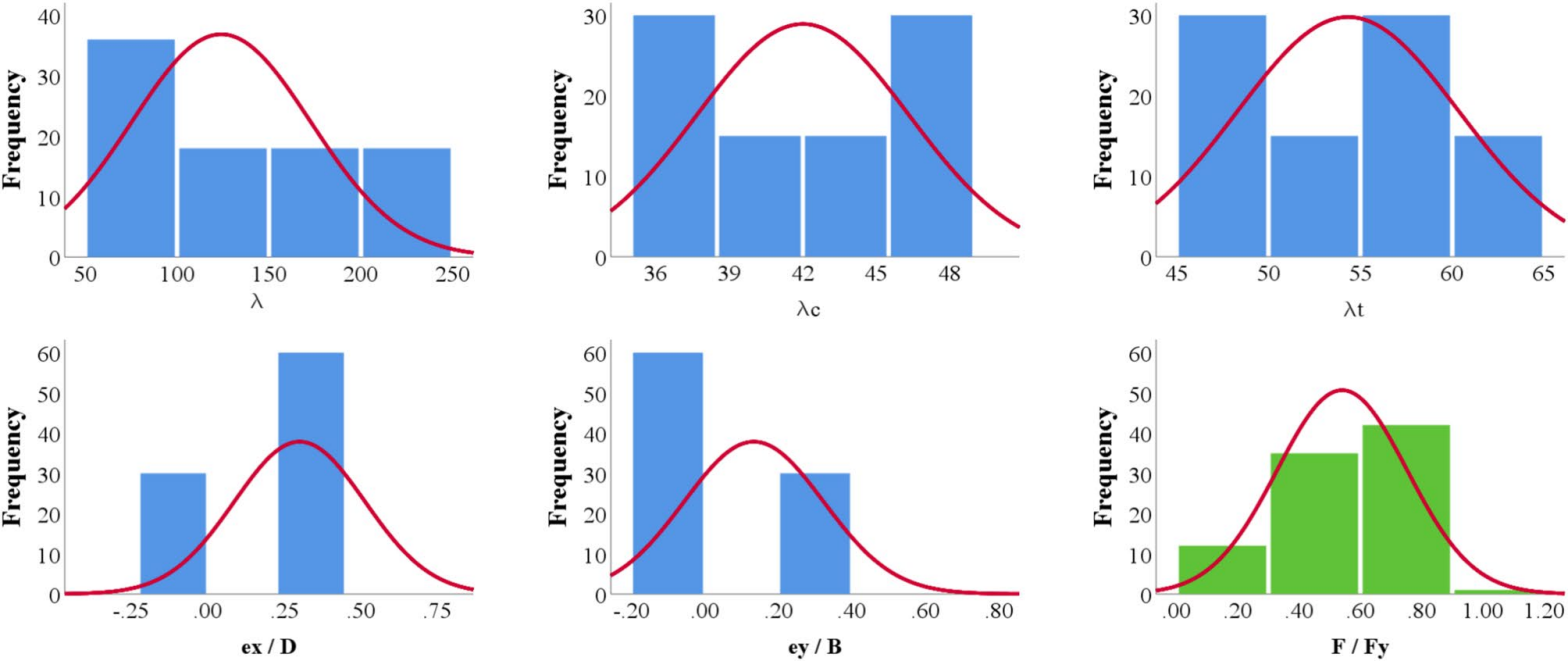
Distribution histograms for inputs (in blue) and outputs (in green).

**Figure 5:**
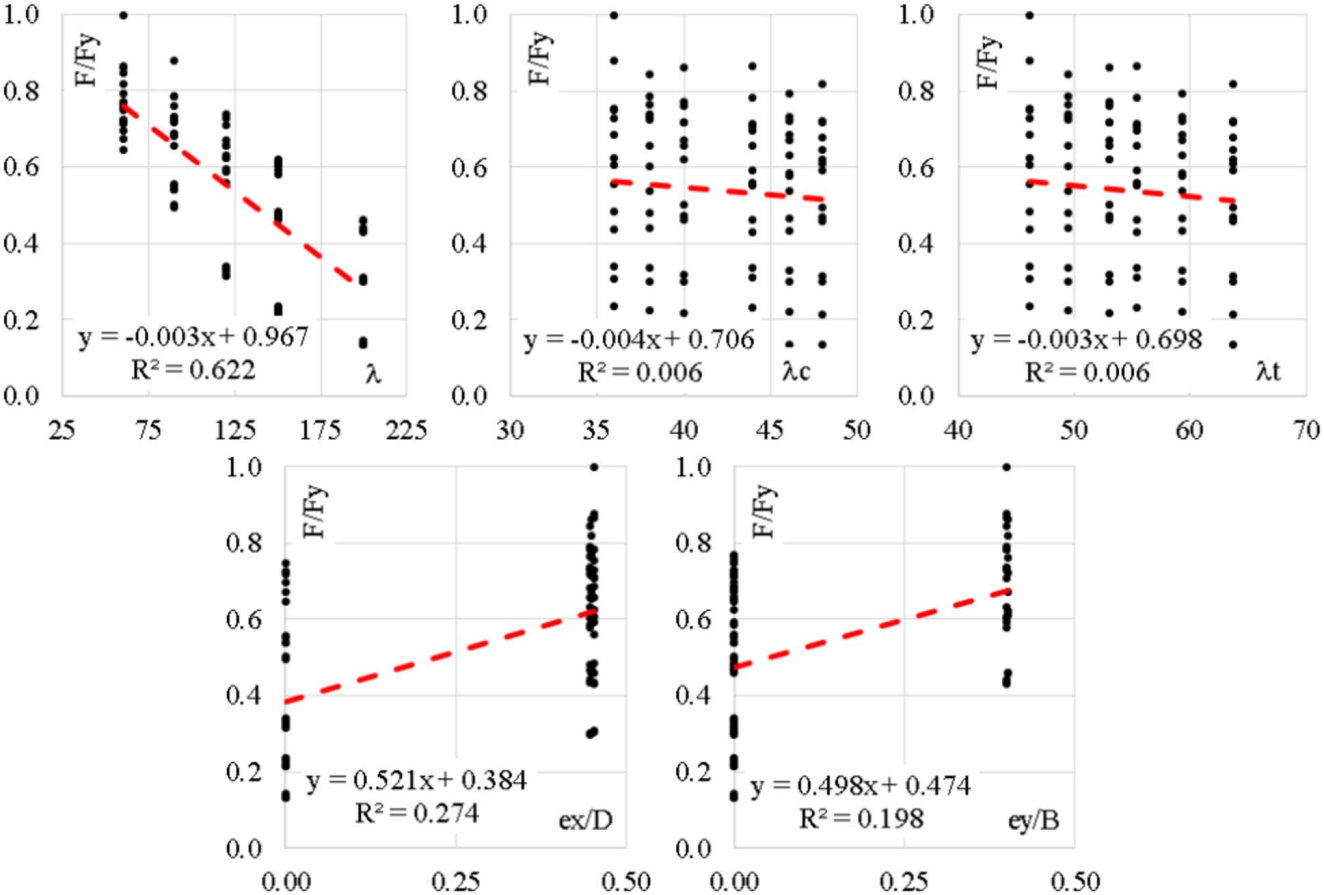
Relations between inputs and output (F/Fy).

Figure 4 shows the histograms for both inputs and outputs, it illustrates the continues probability density function of slenderness ratios and the output and showed gaps in the density function of eccentricities due to lake of test results. On the other hand, Fig. 5 shows the relations between the inputs and the outputs, which indicated a strong inverse relation between the capacity and the global slenderness ratio and moderated forward relation with eccentricities while there is no clear relation with local slenderness ratios.

Table 1 summarizes the statistical characteristics of both training and validation datasets, it assured that they have almost the same range, mean, standard deviation and variations, hence, the validation datasets could be used to monitor the training process and prevents the model over-fitting. Table 2, presents the Pearson correlation matrix, it is used to investigate the interrelations between the considered parameters to make sure that all of them are independent variables and to ranking the inputs according to their impact on the output.

**Table 1:** Statistical analysis of collected database.

	λ	λc	Λt	ex/D	ey/B	F/Fy
Training set						
Max	200.00	48.00	63.70	0.45	0.40	0.88
Min	60.00	36.00	46.17	0.00	0.00	0.13
Avg	122.57	42.28	55.01	0.30	0.14	0.54
SD	46.86	4.48	6.15	0.21	0.19	0.20
Var	0.38	0.11	0.11	0.72	1.38	0.38
Validation set						
Max	200.00	46.06	59.30	0.45	0.40	1.00
Min	60.00	36.00	46.17	0.00	0.00	0.13
Avg	129.00	40.98	52.76	0.32	0.12	0.55
SD	53.19	3.54	4.22	0.21	0.18	0.24
Var	0.41	0.09	0.08	0.65	1.53	0.43

**Table 2:**
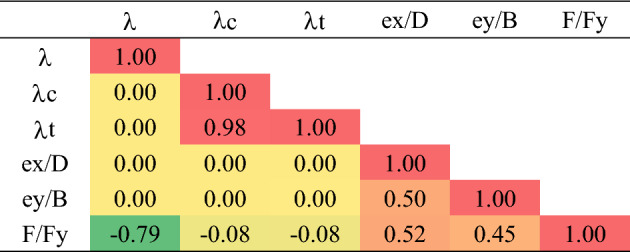
Pearson correlation matrix.

The colors are significant in Table 2

The matrix showed strong correlation between the global slenderness ratio (λ) and the local slenderness ratio of channel (λc), the reason of this correlations is found in the original research program, because as the tested column height increased (λ increased), the size of the used cold form channel increased too, hence (S2) distance increased while the channel thickness is kept constant which means increasing in (λc). Also, the difference between the impact of (λ, λc) on the capacity as shown in the correlation matrix and Fig. 5, assured that they are independent variables.

### Sensitivity analysis

A preliminary sensitivity analysis was carried out on the collected database to estimate the impact of each input on the (F/Fy) values. “Single variable per time” technique is used to determine the “Sensitivity Index” (SI) for each input using Hoffman and Gardener^48^ formula as follows:

7 $$SI \left({X}_{n}\right)= \frac{Y\left({X}_{max}\right)-Y\left({X}_{min}\right)}{Y\left({X}_{max}\right)}$$

The technique depends of measuring the difference in the output values when the value one of the inputs changed from its minimum value to its maximum value while the other inputs kept constants. Applying this technique showed that the (SI) values are (0.53, 0.11, 0.08, 0.34, 0.44) for (λ, λc, λt, ex/D, ey/B) respectively. A sensitivity index of 1.0 indicates complete sensitivity, a sensitivity index less than 0.01 indicates that the model is insensitive to changes in the parameter.

## Results

### GP model

**Figure 6:**
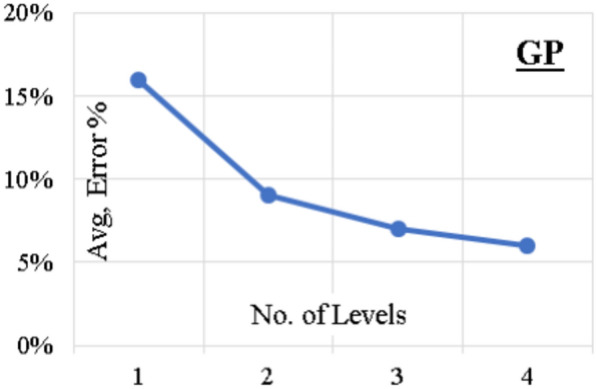
GP model accuracy vs complexity level.

Four GP models were developed with complexity levels ranged between two and five. The population size, survivor size and number of generations were 1000, 300 and 2000, respectively. Figure 6 shows the improvement in accuracy with increasing complexity. Equation (8) presented the output formula for (F/Fy) from the last trial. The average error % of total dataset is (6%), while the (R^2^) value is (0.973).

8 $$\begin {aligned} \frac{{\text{F}}}{{\text{Fy}}}&=\frac{4{{\text{X}}^{4}}}{\left(\lambda {\text{c}}-\lambda \right)}+\frac{{{\text{X}}^{2}}}{3.5\left(\lambda {\text{c}}-\lambda \right)}+\frac{{\text{ey}}}{3.5{\text{B}}}+\text{X}-\frac{1}{102{{\text{X}}^{2}}} \\ {\text{X}}&=\frac{51}{\left(\lambda {\text{c}}-\lambda \right)\left(\frac{{\text{ex}}}{{\text{D}}}\right)+\lambda} \end{aligned}$$

### EPR model

Four developed EPR models were limited to 6th level polynomial, for 5 inputs; there are 462 terms (210 + 126 + 70 + 35 + 15 + 5 + 1 = 462) as follows:

9 $$\begin {aligned} &\sum_{\text{n}=1}^{\text{n}=3}\sum_{\text{m}=1}^{\text{m}=3}\sum_{\text{l}=1}^{\text{l}=3}\sum_{\text{k}=1}^{\text{k}=3}\sum_{\text{j}=1}^{\text{j}=3}\sum_{\text{i}=1}^{\text{i}=3}{{{{{\text{X}}_{\text{n}}.\text{X}}_{\text{m}}.\text{X}}_{\text{l}}.\text{X}}_{\text{k}}.{\text{X}}_{\text{j}}.\text{X}}_{\text{i}}+\sum_{\text{m}=1}^{\text{m}=3}\sum_{\text{l}=1}^{\text{l}=3}\sum_{\text{k}=1}^{\text{k}=3}\sum_{\text{j}=1}^{\text{j}=3}\sum_{\text{i}=1}^{\text{i}=3}{{{{\text{X}}_{\text{m}}.\text{X}}_{\text{l}}.\text{X}}_{\text{k}}.{\text{X}}_{\text{j}}.\text{X}}_{\text{i}} \\ &\quad +\sum_{\text{l}=1}^{\text{l}=3}\sum_{\text{k}=1}^{\text{k}=3}\sum_{\text{j}=1}^{\text{j}=3}\sum_{\text{i}=1}^{\text{i}=3}{{{\text{X}}_{\text{l}}.\text{X}}_{\text{k}}.{\text{X}}_{\text{j}}.\text{X}}_{\text{i}}+\sum_{\text{k}=1}^{\text{k}=3}\sum_{\text{j}=1}^{\text{j}=3}\sum_{\text{i}=1}^{\text{i}=3}{{\text{X}}_{\text{k}}.{\text{X}}_{\text{j}}.\text{X}}_{\text{i}}+\sum_{\text{j}=1}^{\text{j}=3}\sum_{\text{i}=1}^{\text{i}=3}{\text{X}}_{\text{j}}.{\text{X}}_{\text{i}}+\sum_{\text{i}=1}^{\text{i}=3}{\text{X}}_{\text{i}}+\text{C} \end{aligned}$$

**Figure 7:**
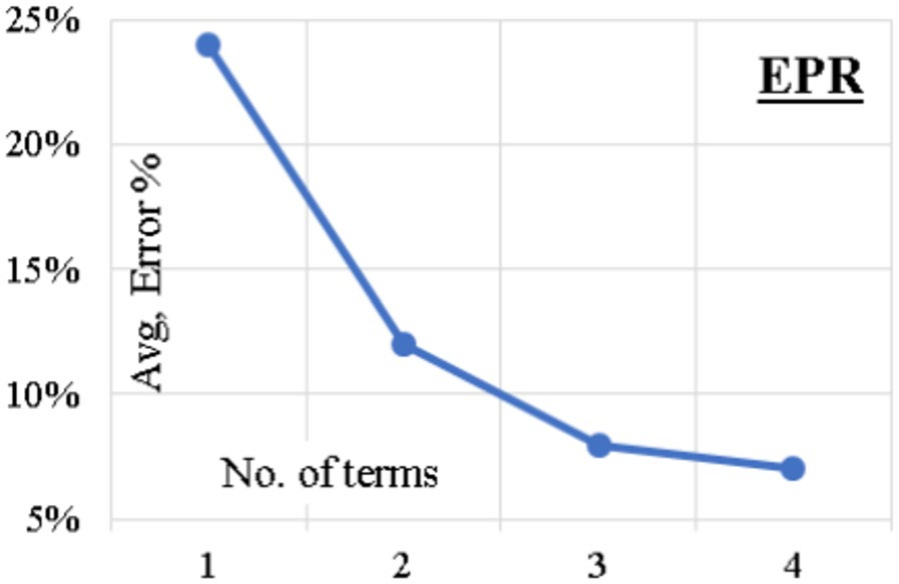
EPR model accuracy vs number of terms.

GA technique was applied on these 462 terms to select the most effective terms to predict the values of (F/Fy). The process began with only 1 term and increased gradually up to 4 terms, Fig. 7 presents the enhancement of fitness with increasing the number of terms and indicates that 4 is the optimum number of terms. The output of the last model is illustrated in Eq. (10). The average error % and (R^2^) values are 7% and 0.965, respectively.

10 $$\frac{F}{Fy} = \left( {\frac{52}{\lambda }} \right) + \left( {\frac{296}{\lambda }} \right)\left( \frac{ex}{D} \right)^{2} - \left( \frac{ex}{D} \right)\left( {\frac{89}{\lambda }} \right)^{2} + \left( \frac{ey}{B} \right)\left( {\frac{772}{{\lambda t}}} \right)^{2} - 0.11$$

### GMDH-NN model

**Figure 8:**
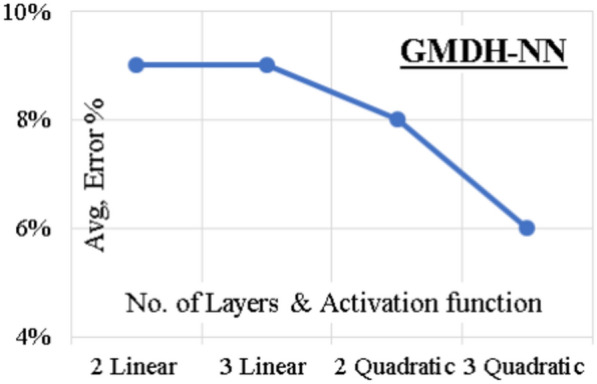
GMDH-NN model accuracy vs number of layer.

Four (GMDH-NN) models were developed to predict (F/Fy) values using “GMDH Shell-3” software. The process included two and three layers with linear activation function and two and three layers with quadratic activation function. The error % values of the four models are illustrated in Fig. 8. The average error % of total dataset is (6%) and the (R^2^) value is (0.979) for the third model. The parametric equation proposed by this model is presented in Eq. (5) where X1 and X2 are substituted from Eqs. (11) and (13).

11 $$\frac{{\text{F}}}{{{\text{Fy}}}}{ } = 0.44{\text{ X}}_{1}^{2} + 0.53{\text{ X}}_{1} { } + { }2.71{\text{ X}}_{2}^{2} - { }2.62{\text{ X}}_{2} + 0.71{ }$$

12 $${\text{X}}_{1} { } = { }\left( {\frac{\lambda }{439}} \right)^{2} - \left( {\frac{\lambda }{167}} \right){ } + \left( {\frac{\lambda }{255}} \right)\left( {\frac{{{\text{ex}}}}{{\text{D}}}} \right){ } + 1.04$$

13 $${\text{X}}_{2} { } = 1.13\left( {\frac{{{\text{ey}}}}{{\text{B}}}} \right) - \left( {\frac{{\lambda {\text{t}}}}{86}} \right)\left( {\frac{{{\text{ey}}}}{{\text{B}}}} \right){ } + 0.47$$

## Discussion

The performances of all the developed models and design codes are summarized in Table 3. The performance of each model was evaluated in terms of SSE, MAE, MSE, RMSE, Error %, Accuracy% and R^2^, for both training and validation datasets. It could be noted that for all models, the performance of both training and validation datasets are almost the same, which assure that the developed models captured the complicated nonlinear relations between parameters without over-fitting. Also, the Tables indicated that both considered design codes (AISI and EC3) exhibited low accuracy because their provisions are based on theoretical analysis and covers a wide range of cold formed steel elements and not limited to composite columns under eccentric load, besides their conservativeness as design codes, they both have almost the same level of accuracy of about 66%. On the other hand, the three developed symbolic regressions also have almost the same level of accuracy of about 94%, this is because they are developed especially for this type of cold formed steel element (track and channel composite column under eccentric load) and using experimental results.

**Table 3:** Accuracies of developed models.

Technique	Dataset	SSE	MAE	MSE	RMSE	Error %	Accuracy%	R^2^
GP	Training	0.085	0.030	0.001	0.035	6	94	0.971
	Validation	0.022	0.027	0.001	0.033	6	94	0.988
EPR	Training	0.101	0.028	0.001	0.038	7	93	0.966
	Validation	0.037	0.033	0.002	0.043	8	92	0.971
GMDH-NN	Training	0.053	0.022	0.001	0.027	5	95	0.982
	Validation	0.032	0.031	0.002	0.040	7	93	0.977
AISI	Training	2.324	0.145	0.033	0.182	34	66	0.699
	Validation	0.786	0.143	0.039	0.198	36	64	0.653
EC3	Training	2.203	0.142	0.031	0.177	33	67	0.724
	Validation	0.660	0.146	0.039	0.182	33	67	0.797

**Figure 9:**
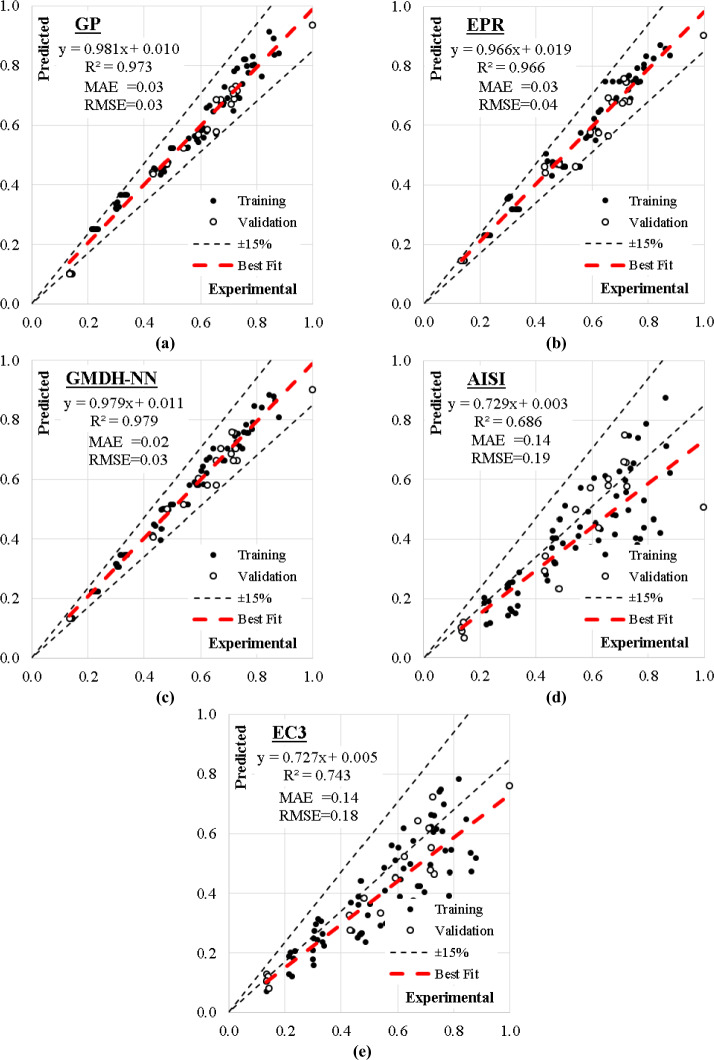
Relation between predicted and calculated (F/Fy) values using the developed models.

The relation between experimental and predicted capacities for the three developed models and the two considered design codes are graphically presented in Fig. 9, where black and white dots are the training and validation datasets respectively, the red dashed line in the best fitting line for the whole dataset and the thin black dashed line presents ± 15% tolerance of the theoretical line (at 45°). The equation of the beat fitting line showed that in average, the predicted values are about 97% and 73% of the experimental ones for the symbolic regression models and design codes in order, while the scattering measurement (R^2^) was about 0.97 and 0.70 for models and codes respectively.

**Figure 10:**
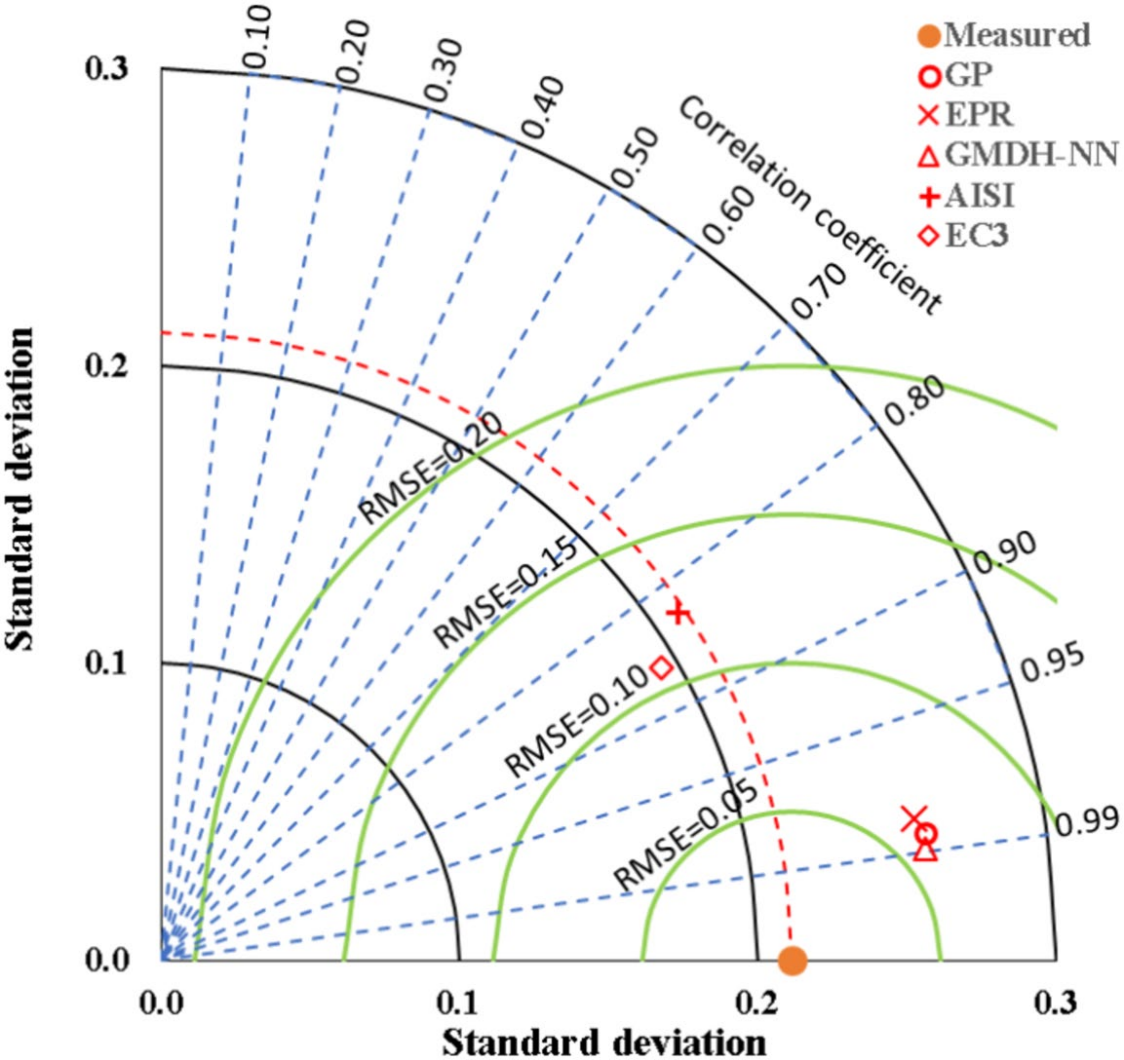
Comparing the accuracies of the developed models using Taylor charts.

Taylor charts in Fig. 10 was used to graphically compare the performance of the both models and codes. The performance of each method (including the experimental method) is presented by a point on the chart based on its RMSE, standard deviation and coefficient of correlation, then the methods could be ranked by their closeness to the experimental point. Usually, coefficient of correlation of 90% is used as acceptance criteria for the developed predictive models, accordingly, the three developed modes are successful. It cloud be also noted that the performance of EC3 is slightly better than AISI.

**Figure 11:**
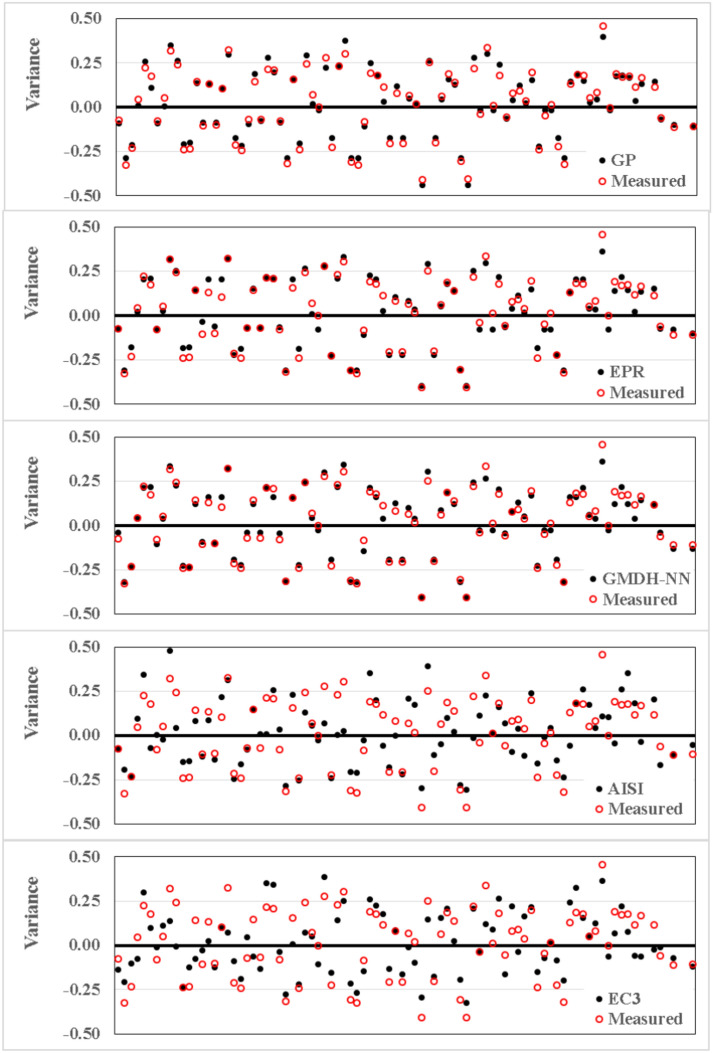
Variance distribution for the developed models.

Finally, the variation of each record for each considered model or code are graphically presented in Fig. 11. Using this figure, the fitness of models and codes could be compared record by record, which allowed to detect and eliminate the outliners (the odd records that have exceptionally large variance for all models).

## Conclusions

This research aims to predict the normalized strength (F/Fy) of bi-axial loaded track and channel cold formed composite column using global and local slenderness ratios (λ, λc, λt) and relative eccentricities in both directions (ex/D, ey/B).Three (AI)-based symbolic regression techniques were used (GP, EPR and GMDH-NN) in addition to two design codes (AISI) and (EC3).The results of comparing the accuracies of the developed models could be concluded in the following points:

- The three predictive models showed the same level of accuracy of 94%, while the two considered design codes also showed the same level of accuracy of 66%. That assured the enhancement of prediction accuracy using the symbolic regression models.
- Both correlation and sensitivity analysis showed that the global slenderness ratio (λ) has the main influence on the strength, then the relative eccentricities (ex/D, ey/B) and finally the local slenderness ratios (λc, λt).
- Both (GP and GMDH-NN) presented an equivalent 4th degree polynomial for the axially loaded normalized strength (ex/D = ey/B = 0), while (EPR) presented much simpler 1st degree invers function which justified the slight reduction in its accuracy.
- Since all the developed models have the same accuracy, it is recommended to use the (EPR) for its simplicity.
- All the developed models are valid only for track and channel cold form composite columns, and they should be verified for other composite columns configurations.
- The developed models are valid within the considered range of parameter values, beyond this range; the prediction accuracy should be verified.
- For farther studies, it is recommend to implement (AI) techniques to predict the behaviour of cold formed columns composed of other sections such as four angles or two sigma sections.

### Supplementary Information

Supplementary Information.

## Data Availability

All data supporting the findings of this study are available within the paper and its Supplementary Information.
