# Supplementary material for: Estimating the strength of bi-axially loaded track and channel cold formed composite column using different AI-based symbolic regression techniques
Source: Sci Rep. 2024 Aug 21;14:19422. doi: 10.1038/s41598-024-69241-6 (PMC11339297; doi:10.1038/s41598-024-69241-6)
Supplement: Supplementary file 1 — Supplementary Information. [file 41598_2024_69241_MOESM1_ESM.docx]

**Appendix (Utilized database)**

| λ | λc | λt | ex/D | ey/B | F/Fy |  | λ | λc | λt | ex/D | ey/B | F/Fy |
| --- | --- | --- | --- | --- | --- | --- | --- | --- | --- | --- | --- | --- |
| Training set | | | | | |  | 150 | 36 | 46 | 0.45 | 0.40 | 0.61 |
| 150 | 46 | 59 | 0.45 | 0.00 | 0.47 |  | 120 | 44 | 55 | 0.45 | 0.00 | 0.56 |
| 150 | 48 | 64 | 0.00 | 0.00 | 0.21 |  | 200 | 48 | 64 | 0.00 | 0.00 | 0.13 |
| 200 | 44 | 55 | 0.45 | 0.00 | 0.31 |  | 60 | 46 | 59 | 0.45 | 0.40 | 0.79 |
| 120 | 46 | 59 | 0.45 | 0.00 | 0.59 |  | 120 | 36 | 46 | 0.00 | 0.00 | 0.34 |
| 60 | 38 | 49 | 0.45 | 0.00 | 0.76 |  | 150 | 38 | 49 | 0.45 | 0.40 | 0.60 |
| 60 | 48 | 64 | 0.45 | 0.00 | 0.72 |  | 120 | 36 | 46 | 0.45 | 0.40 | 0.73 |
| 200 | 40 | 53 | 0.45 | 0.40 | 0.46 |  | 90 | 48 | 64 | 0.45 | 0.00 | 0.68 |
| 120 | 48 | 64 | 0.45 | 0.00 | 0.59 |  | 150 | 36 | 46 | 0.00 | 0.00 | 0.24 |
| 60 | 40 | 53 | 0.45 | 0.40 | 0.86 |  | 200 | 46 | 59 | 0.00 | 0.00 | 0.13 |
| 90 | 44 | 55 | 0.45 | 0.40 | 0.78 |  | 90 | 40 | 53 | 0.45 | 0.40 | 0.76 |
| 200 | 40 | 53 | 0.45 | 0.00 | 0.30 |  | 90 | 40 | 53 | 0.00 | 0.00 | 0.50 |
| 200 | 36 | 46 | 0.45 | 0.00 | 0.31 |  | 90 | 36 | 46 | 0.45 | 0.40 | 0.88 |
| 90 | 46 | 59 | 0.45 | 0.00 | 0.68 |  | 90 | 44 | 55 | 0.00 | 0.00 | 0.55 |
| 200 | 36 | 46 | 0.45 | 0.40 | 0.44 |  | 90 | 48 | 64 | 0.45 | 0.40 | 0.72 |
| 60 | 46 | 59 | 0.00 | 0.00 | 0.67 |  | 150 | 36 | 46 | 0.45 | 0.00 | 0.48 |
| 200 | 38 | 49 | 0.45 | 0.40 | 0.44 |  | 150 | 40 | 53 | 0.45 | 0.40 | 0.62 |
| 60 | 48 | 64 | 0.00 | 0.00 | 0.65 |  | 120 | 46 | 59 | 0.45 | 0.40 | 0.63 |
| 60 | 44 | 55 | 0.45 | 0.40 | 0.86 |  | 150 | 46 | 59 | 0.45 | 0.40 | 0.58 |
| 120 | 46 | 59 | 0.00 | 0.00 | 0.33 |  | 120 | 38 | 49 | 0.45 | 0.40 | 0.74 |
| 200 | 46 | 59 | 0.45 | 0.00 | 0.30 |  | 200 | 48 | 64 | 0.45 | 0.00 | 0.30 |
| 150 | 48 | 64 | 0.45 | 0.00 | 0.47 |  | 90 | 48 | 64 | 0.00 | 0.00 | 0.49 |
| 90 | 36 | 46 | 0.45 | 0.00 | 0.69 |  | 90 | 36 | 46 | 0.00 | 0.00 | 0.56 |
| 150 | 40 | 53 | 0.45 | 0.00 | 0.47 |  | 120 | 40 | 53 | 0.00 | 0.00 | 0.32 |
| 60 | 36 | 46 | 0.45 | 0.00 | 0.75 |  | 150 | 46 | 59 | 0.00 | 0.00 | 0.22 |
| 60 | 36 | 46 | 0.00 | 0.00 | 0.75 |  | Validation set | | | | | |
| 150 | 44 | 55 | 0.45 | 0.00 | 0.46 |  | 120 | 40 | 53 | 0.45 | 0.40 | 0.67 |
| 150 | 38 | 49 | 0.00 | 0.00 | 0.22 |  | 60 | 38 | 49 | 0.00 | 0.00 | 0.72 |
| 60 | 44 | 55 | 0.00 | 0.00 | 0.70 |  | 60 | 46 | 59 | 0.45 | 0.00 | 0.72 |
| 200 | 38 | 49 | 0.45 | 0.00 | 0.30 |  | 150 | 44 | 55 | 0.45 | 0.40 | 0.59 |
| 90 | 38 | 49 | 0.45 | 0.40 | 0.78 |  | 120 | 36 | 46 | 0.45 | 0.00 | 0.62 |
| 150 | 48 | 64 | 0.45 | 0.40 | 0.61 |  | 60 | 36 | 46 | 0.45 | 0.40 | 1.00 |
| 90 | 38 | 49 | 0.00 | 0.00 | 0.54 |  | 90 | 46 | 59 | 0.00 | 0.00 | 0.54 |
| 60 | 48 | 64 | 0.45 | 0.40 | 0.82 |  | 90 | 38 | 49 | 0.45 | 0.00 | 0.73 |
| 120 | 48 | 64 | 0.00 | 0.00 | 0.32 |  | 60 | 44 | 55 | 0.45 | 0.00 | 0.71 |
| 60 | 40 | 53 | 0.45 | 0.00 | 0.77 |  | 90 | 40 | 53 | 0.45 | 0.00 | 0.72 |
| 60 | 38 | 49 | 0.45 | 0.40 | 0.84 |  | 120 | 38 | 49 | 0.45 | 0.00 | 0.66 |
| 150 | 44 | 55 | 0.00 | 0.00 | 0.23 |  | 120 | 44 | 55 | 0.45 | 0.40 | 0.71 |
| 150 | 40 | 53 | 0.00 | 0.00 | 0.22 |  | 200 | 44 | 55 | 0.00 | 0.00 | 0.14 |
| 200 | 48 | 64 | 0.45 | 0.40 | 0.46 |  | 90 | 44 | 55 | 0.45 | 0.00 | 0.66 |
| 90 | 46 | 59 | 0.45 | 0.40 | 0.73 |  | 150 | 38 | 49 | 0.45 | 0.00 | 0.48 |
| 60 | 40 | 53 | 0.00 | 0.00 | 0.72 |  | 200 | 38 | 49 | 0.00 | 0.00 | 0.14 |
| 120 | 40 | 53 | 0.45 | 0.00 | 0.66 |  | 200 | 44 | 55 | 0.45 | 0.40 | 0.43 |
| 120 | 38 | 49 | 0.00 | 0.00 | 0.34 |  | 200 | 36 | 46 | 0.00 | 0.00 | 0.14 |
| 120 | 48 | 64 | 0.45 | 0.40 | 0.62 |  | 200 | 40 | 53 | 0.00 | 0.00 | 0.13 |
| 120 | 44 | 55 | 0.00 | 0.00 | 0.33 |  | 200 | 46 | 59 | 0.45 | 0.40 | 0.43 |
